# Identification of heat-responsive genes in carnation (*Dianthus caryophyllus* L.) by RNA-seq

**DOI:** 10.3389/fpls.2015.00519

**Published:** 2015-07-14

**Authors:** Xue Li Wan, Qiao Zhou, Yuan Yuan Wang, Wen En Wang, Man Zhu Bao, Jun Wei Zhang

**Affiliations:** Key Laboratory of Horticultural Plant Biology, Ministry of Education, College of Horticulture and Forestry Sciences, Huazhong Agricultural UniversityWuhan, China

**Keywords:** heat stress, transcriptome, expression profiling, candidate genes, carnation

## Abstract

Carnation (*Dianthus caryophyllus* L.) is an important flower crop, having substantial commercial value as a cut-flower due to the long vase-life and wide array of flower colors and forms. Standard carnation varieties perform well under cool climates but are very susceptible to high temperatures which adversely affect the yield and the quality of the cut-flowers. Despite several studies of carnation contributing to the number of expressed sequence tags (ESTs), transcriptomic information of this species remains very limited, particularly regarding abiotic stress-related genes. Here, transcriptome analysis was performed to generate expression profiles of heat stress (HS)-responsive genes in carnation. We sequenced a cDNA library constructed with mixed RNA from carnation leaves subjected to 42°C HS (0, 0.5, 1, and 2 h) and 46°C HS (0.5, 1, and 2 h), and obtained 45,604,882 high quality paired-end reads. After *de novo* assembly and quantitative assessment 99,255 contigs were generated with an average length of 1053 bp. We then obtained functional annotations by aligning contigs with public protein databases including NR, SwissProt, KEGG, and COG. Using the above carnation transcriptome as the reference, we compared the effects of high temperature treatments (42°C: duration 0.5, 2, or 12 h) delivered to aseptic carnation seedlings, relative to untreated controls, using the FPKM metric. Overall, 11,471 genes were identified which showed a significant response to one or more of the three HS treatment times. In addition, based on GO and metabolic pathway enrichment analyses, a series of candidate genes involved in thermo-tolerance responses were selected and characterized. This study represents the first expression profiling analysis of *D. caryophyllus* under heat stress treatments. Numerous genes were found to be induced in response to HS, the study of which may advance our understanding of heat response of carnation.

## Introduction

Carnation (*Dianthus caryophyllus* L.) is one of the most important flowers in the global floriculture industry. The carnation species is best adapted to cool climates, with the optimum temperature for growth and flowering being between 13 and 15°C during the summer months and 10–11°C during winter. High temperatures are generally detrimental to the growth and development of carnation plants (Lim, 2014). However, studies of carnation are typically focused on maximizing cut-flower quality and longevity, or the physiological and biochemical aspects of the plant's response to the environment (In et al., 2013). To date, very little data is available regarding the heat response mechanism of carnation.

In recent years, the threat of global warming and the wide-reaching implications of the adverse effects on plant growth have encouraged the study of heat stress (Cramer et al., 2011; Bokszczanin et al., 2013). High temperatures typically stimulate the expression of heat shock protein genes (HSPs), and these can have key roles in enabling the survival of an individual in the face of potentially fatal high temperatures. HSPs are known to act as chaperones in protein folding in order to preserve protein stability and functionality when high temperatures would otherwise induce protein denaturation. Members of the family of heat shock transcription factors (HSFs) play key roles in the signaling pathways that are triggered by heat stress, and are ultimately involved in the regulation of downstream targets including HSPs. However, cellular regulation triggered by heat stress consists of multiple and interconnected signal transduction pathways stretching from the initial perception to the final thermo-tolerance response. In *Arabidopsis*, abscisic acid (ABA), salicylic acid (SA), ethylene, oxidative burst, galactinol synthase (GolS), multi-protein bridging factor 1c (MBF1c), and calmodulin are all known to play significant roles in the heat stress response (Rizhsky et al., 2004; Busch et al., 2005; Volkov et al., 2006; Suzuki et al., 2008; Miller et al., 2010; Pillet et al., 2012). In carnation, however, no gene associated with the thermo-tolerance response has, to date, been the subject of a detailed investigation.

Currently, transcriptomic studies are widely used to systematically investigate the molecular reactions by which plants respond and adapt to complicated environmental changes. By high throughput microarray analyses, wide spectrums of genes have been shown to respond to HS in *Arabidopsis* (Rizhsky et al., 2004; Busch et al., 2005; Swindell et al., 2007; Larkindale and Vierling, 2008). By applying a similar approach, HS regulated genes have been identified in rice (Zhang et al., 2012; Sarkar et al., 2014), tomato (Frank et al., 2009; Bita et al., 2011), potato (Ginzberg et al., 2009), and grape (Liu et al., 2012; Carbonell-Bejerano et al., 2013). Despite the wealth of recently published data regarding HS mechanisms in several crop and model plant systems, little or no information is available regarding this response in the ornamental crop plant species. Recently, the mining of genes relating to cut-flower longevity was enabled by transcriptome analysis in carnation (Tanase et al., 2012). Subsequently, Yagi et al. (2014) released the genome sequence of carnation, thereby providing a valuable data resource for further research in this species. However, the genetic basis of the physiological and molecular response of carnation to high temperatures has not yet been investigated.

In this study, a *de novo* transcriptome was generated using a cDNA library constructed from carnation leaves subjected to heat stress. By using this transcriptome as the reference set of sequences, we performed expression profiling analysis of carnation by RNA-Seq in response to a time course of HS exposure (0, 0.5, 2, and 12 h). Expression changes in response to the HS treatment were confirmed for a subset of the genes (these genes were chosen to represent five groups of notable differentially regulated genes that were considered likely to be involved in the HS response of carnation leaves, i.e., HSFs, HSPs, ROS-scavenging enzymes and genes related to signal transduction and carbohydrate metabolism) by quantitative Real-Time PCR (qRT-PCR) analysis. Our goal was to identify candidate genes with altered levels of transcript accumulation in carnation leaves subjected to HS. In particular, we were interested in genes potentially involved with metabolic regulation during early and subsequent stages of the cellular HS response. We speculate that such genes may reflect the importance of transcriptional regulation, reactive oxygen species (ROS) scavengers, signal transduction, and metabolism as essential components of the HS response in carnation.

## Results

### Basic statistical analysis of transcriptome sequencing

In this study, we constructed a non-normalized cDNA library using an equally mixed pool of seven mRNA populations which had been extracted from leaf tissues subjected to various heat stress treatments (i.e., 42°C: 0, 0.5, 1, 2 h; 46°C: 0.5, 1, 2 h). The library was sequenced on the Illumina Hiseq2000 platform using the paired end protocol. Filtering and conversion of raw reads to FASTQ format resulted in 45,604,882 paired-end reads with lengths of at least 2 × 100 nucleotides (Table 1).

**Table 1:** **Summary of *de novo* transcriptome assembly**.

**Items**	**Numbers**
Filtered reads (paired-ends)	45,604,882
Filtered reads (single-end)	91,209,764
Total assemble size (bp)	104,521,408
Number of contigs	99,255
Average length (bp)	1053
Longest contig (bp)	15,454
Shortest contig (bp)	201
N50 (bp)	1696
GC content (%)	42

*De novo* assembly of the filtered reads data was performed using Trinity. This analysis identified 99,255 contigs with an N50 gene size of 1696 bp, a maximum contigs length of 15,454 bp, and an average length of 1053 bp (Table 1). The transcriptome data have been deposited to Sequence Read Archive (SRA) and the accession number is SRR2025071.

### Annotation and functional classification of transcriptome sequencing

The annotation of the transcriptome sequences was based on the sequence homology outcomes of BLASTX search against the public protein databases. Search results aligned 50% of the contigs with known proteins and their putative functions, while the remaining 50% showed various levels of homology with unknown or hypothetical proteins having no characterized function.

**Figure 1:**
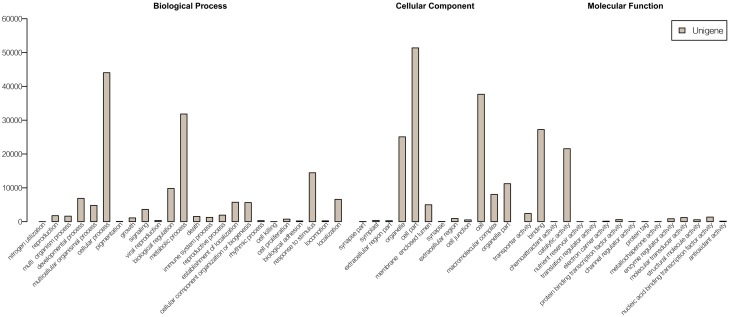
**GO annotations of non-redundant consensus sequences**. Best hits were aligned to the GO database, and most consensus sequences were grouped into three major functional categories namely, biological process, cellular component, and molecular function.

The unigene sequences were further characterized by the assignment of gene ontology (GO) terms (Figure 1). The most highly represented GO categories of biological processes were those of cellular processes (44,042 unigenes) and metabolic processes (31,829 unigenes), suggesting a high degree of basic metabolic activity in the heat-stressed tissues. Important GO slim categories, but with lower numbers of unigene alignments, were those of response to stimulus (14,448 unigenes), signal transduction (3631 unigenes), nucleic acid binding transcription factor activity (1352 unigenes) and transporter activity (2407 unigenes). Each of these categories has been implicated in general stress response pathways, consisting of stress recognition, downstream signaling events, and defense and adaptation responses.

### RNA-seq quality control analysis

Samples from three biological replicates of four treatments namely, heat stress at 42°C delivered for 0 h (HS_0h), 0.5 h (HS_0.5h), 2 h (HS_2h), and 12 h (HS_12h) were sequenced for transcript profiling. Gene-body coverage, numbers of splicing junctions and nucleotide frequency of the data were tested. Additionally, the numbers of reads and total mapped reads were analyzed (Additional file 1). The results showed a high mapping rate and good gene-body coverage. Therefore, we judged our RNA-seq data to be of sufficiently high quality to merit further investigation. The RNA-Seq data have been deposited to Sequence Read Archive (SRA) and the accession number is SRR2027757.

### Differentially expressed genes in response to HS

**Figure 2:**
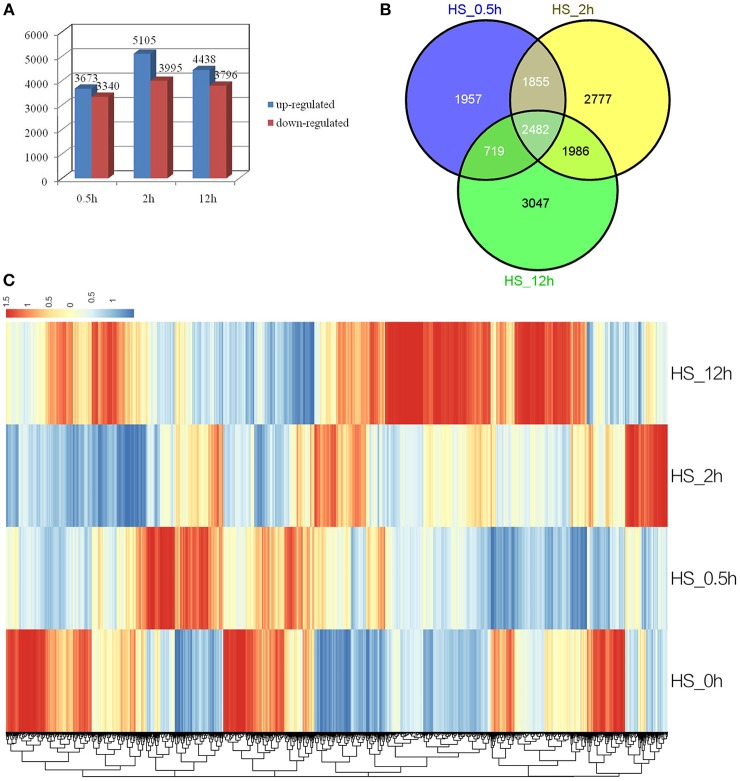
**Analysis of differentially expressed genes in response to HS. (A)** Numbers of the significantly regulated genes at the three HS treatment time points. The numbers on the horizontal axis represent the three time points while, the vertical axis reflects the numbers of up-regulated and down-regulated genes. Up- and down-regulated genes are shown in blue and red bars, respectively. **(B)** Venn diagram analysis of the significantly regulated genes at the three HS treatment time points. **(C)** Heatmap clustering of global pattern of the significantly regulated genes conducted using Hierarchical Clustering (HCL) algorithm at the three HS treatment time points. The color scale represents the values of lg FPKM (FPKM – Fragments Per Kilobase of transcript per Million fragments mapped).

To identify genes with altered expression levels under conditions of heat shock, the mRNA profile of control plants (HS_0h) was compared with those of plants treated with HS_0.5h, HS_2h, and HS_12h. According to the criteria of twofold up- or down-regulation, 11,471 genes were identified as differentially-regulated genes (DEGs) in at least one of the conditions in this study. Analysis of DEGs revealed that 3673 genes were rapidly upregulated (*P* < 0.05) at HS_0.5h as compared to 3340 down-regulated genes. The number of DEGs reached a peak at HS_2h and was reduced at HS_12h (Figure 2A). A total of 2482 genes were found to show significantly altered expression levels at all three of the HS time points, as compared to the control (Figure 2B).

Strongly up-related genes (Log2FC ≧ 4) and down-related genes (Log2FC ≦ −4) were identified from expression profile analysis at the three HS treatment time points (Additional file 2, Additional file 3). These genes mostly comprised stress response HSPs, transcription factors, ROS-related genes and signal transduction factors. Genes encoding heat shock proteins (HSPs) which were the most highly up-regulated under heat stress included *Dc_25002*, annotated as cytoplasmic class I small heat shock protein and expression of which was 256-fold higher at HS_0.5h and HS_2h than in the 0h control, and *Dc_83420*, putative heat shock transcription factor A2, the Log2FC expression value of which reached approximately 9.6 at HS_0.5h.

A Hierarchical Clustering (HCL) algorithm was used to generate a heat map visualization of the global clustering pattern of the significantly regulated genes (Figure 2C). This revealed two major patterns of response. Thus, the majority of strongly up-related genes (Log2FC ≧ 4) were seen to have achieved the highest transcriptional level at HS_0.5h or HS_2h, and showed a relatively low level of expression at HS_12h; this pattern included the HSPs. Conversely, some strongly down-related genes (Log2FC ≦ −4) showed the most significant expression change at HS_12h; this pattern included some metabolism genes (Figure 2C, Additional file 2, Additional file 3).

### Real-time quantitative PCR analysis

**Figure 3:**
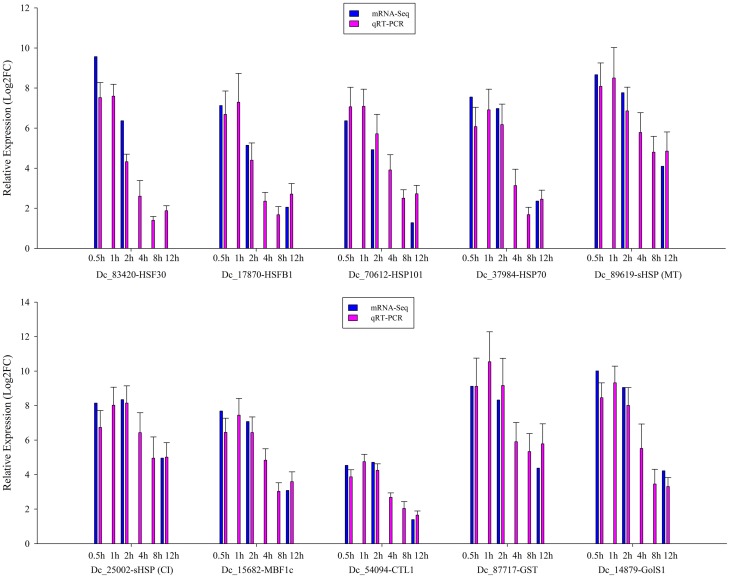
**qRT-PCR verification of the differentially expressed genes**. Error bars indicate standard deviation. Log2FC: fold change value after log2 transformation of expression levels.

**Figure 4:**
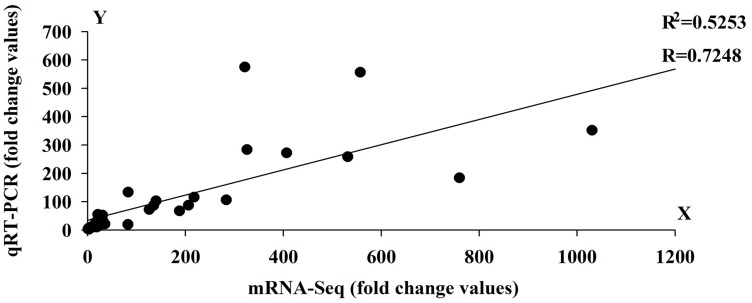
**Linear regression analysis between Quantitative Real-Time PCR (qRT-PCR) and RNA-seq results (*r* = 0.7248) for 10 genes**. X-axis numbers represent the fold change values of mRNA-seq results. Y-axis numbers represent the fold change values of qRT-PCR results.

To validate the expression profile data, 10 genes with significant expression changes were examined by real-time quantitative PCR (qRT-PCR) using gene-specific primers (Additional file 4). In addition to the three time points (HS_0.5h, HS_2h, HS_12h) as analyzed by mRNA-Seq, three further time points (HS_1h, HS_4h, HS_8h) were also analyzed by qRT-PCR (Figure 3). All 10 transcripts showed similar expression pattern in this analysis as seen from the *in silico* differential analysis results from high-throughput sequencing (Figure 4).

### Gene ontology analysis of the significantly regulated genes

**Figure 5:**
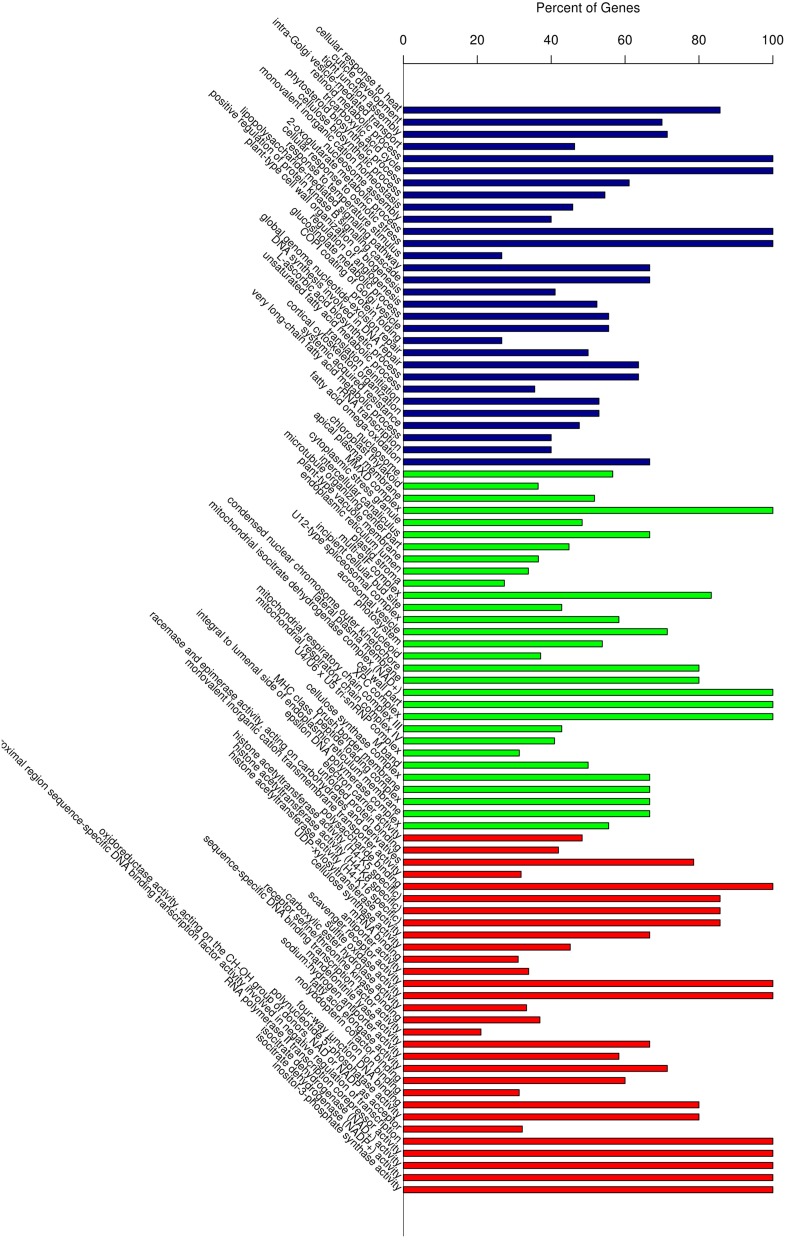
**Gene Ontology (GO) enrichment analysis of all the differentially expressed genes**. Vertical axis displays the percentage of significant genes corresponding to each functional type. Horizontal axis displays the GO annotation corresponding to the three categories: from left to right: BP, CC, and MF, represented by colors: blue, green, and red), respectively. BP, Biological Process; CC, Cellular Compartment; MF, Molecular Function.

Gene Ontology (GO) classification of the significantly regulated genes was performed to identify the functional processes associated with the carnation leaf HS response. The predominant 30 sub-classifications of GO functions across all of the significantly regulated genes, as determined over the three HS treatment time points, is shown in Figure 5. Based on these functional classifications, we compared the significantly regulated genes at each of the three time points (Additional file 5). Overall, these regulated genes were mainly related to abiotic stimulus responses, signaling and metabolic activity and transport categories. The expression changes of some genes, including the HSPs response to stimulus (GO:0009628), showed a combination of differences and similarities between the three time points. A subset of regulated genes showed a reversal of the expression response over the HS time period thus, some were up-regulated in response to HS_0.5h and down-regulated following HS_12h whereas, other genes showed the reverse pattern. Another subset of regulated genes showed either significant differences or no significant differences between the expression levels at each of the time points. The gene group for response to abiotic stimulus (GO:0009628) illustrates these diverse temporal response patterns thus, in this GO group there were identified 158, 187, and 186 significantly regulated genes at the treatment time points HS_0.5h, HS_2h, and HS_12h, respectively (Additional file 6). In this group, 43 genes showed no significant differences in expression levels when compared at HS_0.5h whereas, >70 genes did show significant expression differences at this time point. Moreover, 45 further genes were significantly regulated at both HS_0.5h and HS_2h time points. These observations demonstrate that diverse gene expression patterns define the cellular response to heat stress.

### HSPs in response to HS

From the list of significantly regulated genes, 44 represented highly up-related HSPs (Log2FC ≧ 4 on at least one HS time point). Of these, 15 genes represented various types of small HSP (cytosolic class I, II, and III, as well as chloroplast, endoplasmic reticulum and mitochondrial), 18 genes coded for HSP70 forms (cytosolic, chloroplast, endoplasmic reticulum and mitochondrial), 3 for HSP90, and 2 for HSP101. In addition, 6 genes coded for DNAJ. Most of these genes exhibited their highest transcriptional levels at HS_2h, with decreased expression seen at HS_12h, although, the latter levels were still markedly higher than in the untreated control samples (Additional file 2).

### Effects of HS on transcription factors

The results of this study showed that TFs of different families were significantly regulated in response to HS, and the most strongly up- or down-regulated TFs (Log2FC ≧4/≦ −4) predominately belonged to HSF, AP2/ERF, MYB, NAC, bHLH, and bZIP families (Additional file 2, Additional file 3). Among these HS-responsive TFs, the *Hsf* genes exhibited the greatest change in expression levels. At HS_0.5h, there were seen to be 8 significantly differentially regulated *Hsf* members (i.e., 1 gene for *Hsf A1*, 3 for *HsfA2*, 3 for *HsfB1*, and 1 for *HsfC1*). Among these, *Dc_83420* (*HsfA2*) and *Dc_17870* (*HsfB1*) showed the most notable up-regulation of expression with Log2FC values of 9.569 and 7.126, respectively. By contrast, *Dc_52566* (*HsfC1*) was strongly down-regulated. With regard to expression responses at the later HS time points, 5 *Hsf* members (2 genes for *HsfA2*, 1 for *HsfA3*, and 2 for *HsfB1*) were upregulated at HS_2h, while 6 *Hsf* members (1 gene for *HsfA2*, 1 for *HsfA3*, 3 for *HsfB1*, and 1 for *HsfC1*) were up-regulated at HS_12h (Additional file 7).

### ROS-scavenging genes in response to HS

**Figure 6:**
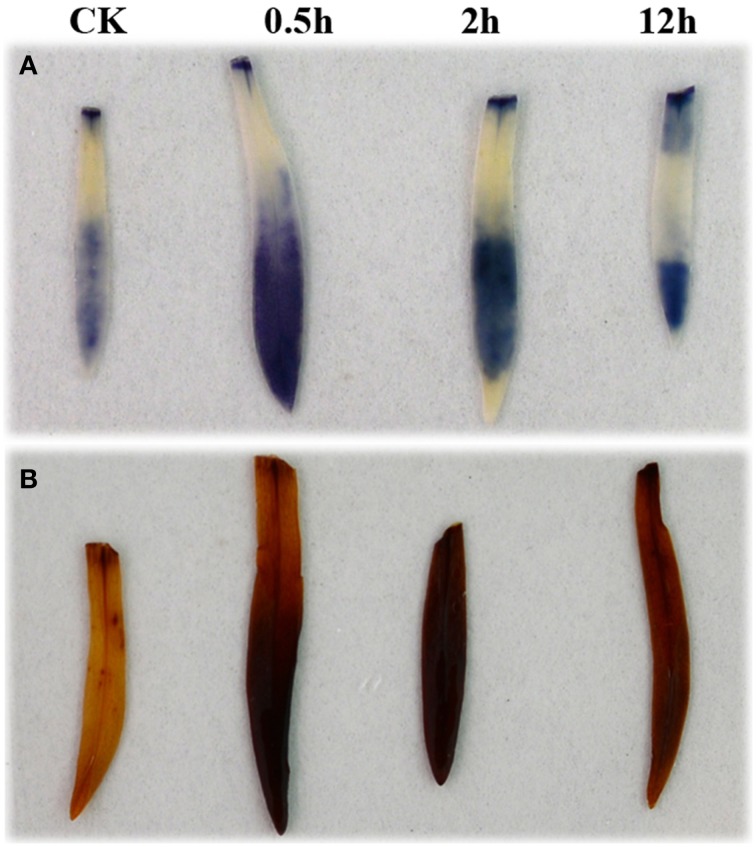
**Accumulation of superoxide anion and hydrogen peroxide in carnation leaves under heat shock. (A)** Accumulation of superoxide anion. **(B)** Accumulation of hydrogen peroxide. Plants were treated at 42°C and leaves were collected at 0 h (as a control), 0.5, 2, and 12 h. Immediately following collection of the heat-treated leaves, samples were immersed in NBT solution to detect superoxide anion, or DAB solution to detect hydrogen peroxide, and incubated in darkness for 3 or 24 h, respectively.

The transcript group associated with the GO term of reactive oxygen species (GO:0000302) was enriched at each of the three HS treatment time points, and several genes involved in the production or scavenging of ROS were upregulated in samples from at least one of the treatment times. *APX1* (*Dc_69354*) was found to be up-regulated at HS_0.5h and HS_2h, but had returned to control levels by HS_12h. Three genes encoding superoxide dismutase copper chaperones showed significant responses to the HS_2h treatment, with two (*Dc_17123* and *Dc_89779*) being up-regulated, and one (*Dc_4138*) down-regulated. Mitogen-Activated Protein Kinase 3 (*Dc_51188*) was down-regulated at HS_2h whereas, E3 ubiquitin-protein ligase UPL5 (*Dc_5771*) was up-regulated at the same time point (Additional file 8). We analyzed those ROS-related genes which showed the strongest levels of up- or down-regulation (Log2FC ≧4/≦−4) across the HS treatments. This revealed that the majority of highly up-regulated ROS genes responded relatively rapidly, i.e., within the first 2 h of HS; this group included *APX2, FKB70, GST*, and *AOX1* (Additional file 2). By contrast, most of the strongly down-regulated ROS genes did not show a significant response until late into the treatment, i.e., HS_12h, and *APX3* is an example of this (Additional file 3). In an attempt to equate these gene expression changes with the presence of the major ROS, we visualized the accumulation of superoxide (O^−^_2_) and hydrogen peroxide (H_2_O_2_) during the heat treatment of carnation leaves using nitroblue tetrazolium (NBT) and 3,3′-diaminobenzidine tetrahydrochloride (DAB), respectively. The results showed that these ROS were produced as part of the initial heat shock response, but levels had decreased by the later stages of the heat shock process (Figure 6).

### Signal transduction

From GO analysis it was seen that the transcript group associated with the signaling functional term (GO:0023052) was enriched at all three of the HS time points, and 66 genes involved in signal transduction were found to show significant responses. Of these genes, multiprotein-bridging factor 1c (*MBF1c*) (*Dc_15682*) was found to show the highest degree of up-regulation, and expression of calreticulin was also substantially elevated (Additional file 9). In contrast, receptor protein kinase CLAVATA, serine/threonine-protein kinase BRI1-like, leucine-rich repeat transmembrane protein kinase, and Ras GTPase-activating protein-binding protein 1 were down-regulated in the HS-treated tissues.

Of the 31 signaling-related genes that showed an early response to heat treatment (i.e., at HS_0.5h), the homeobox-leucine zipper protein ATHB-6 gene (*Dc_2661*) showed a relatively weak down-regulation effect, while the early response of ethylene receptor 2 gene (*Dc_89516*) was in the opposite direction. A total of 57 signaling-related genes, for example, ethylene receptor 2 (*Dc_21144*), showed a significant change in expression at HS_12h. The auxin-responsive protein *IAA9* (*Dc_43493*) was found to be up-regulated at HS_2h and this elevated expression persisted at HS_12h (Additional file 9). *ERF5* (*Dc_24004*) and *ERF10* (*Dc_58906*) were down-regulated in response to HS whereas, *ERF12* (*Dc*_91040) was up-regulated. Other signaling-related genes up-regulated by HS included ADP-ribosylation factor GTPase-activating protein AGD11 (*Dc_53115*), calreticulin (*Dc_91700*), *MBF1c* (*Dc_15682*), and Ras-related protein Rab7 (Additional file 2). Signaling-related genes that were down-regulated by HS included 5 auxin-related genes and 3 G2/mitotic-specific cyclin genes (Additional file 3).

### Involvement of carbohydrate metabolism in HS response

We analyzed the HS-regulated genes corresponding to the GO category of carbohydrate metabolic process (GO:0005975) (Additional file 10). Among these genes, sucrose-related genes such as, Inositol-3-phosphate synthase (*Dc_34653*), glycosyltransferase (*Dc_61803*), cellulose synthase A catalytic subunit 3 (*Dc_78418*) and sucrose synthase (*Dc_8940, Dc_91245*) were up-regulated, while trehalose-phosphate synthase (*Dc_25550*), UDP-glucuronic acid decarboxylase (*Dc_61406*), flavonol 4′-sulfotransferase (*Dc_69704*), and alpha-xylosidase (*Dc_97561*) were down regulated.

Metabolism-related genes with a strong HS-response included GolS1 (*Dc_14879*) which showed an expression increase corresponding to a Log2FC value of approx. 10, and this was seen within the first 2 h of heat treatment (Additional file 2). In contrast, the majority of strongly down-regulated genes relating to metabolism showed a maximal response at HS_12h (Additional file 3).

## Discussion

### Sequence analysis of transcriptome and genome of carnation

Tanase et al. (2012) have published the transcriptome from carnation cultivar “Francesco,” which comprised 300,740 genes. Two years later, a whole genome sequence was made available (Yagi et al., 2014). Transcriptome analysis of *D. caryophyllus* “Francesco” identified 37,844 ESTs, and this compares to 56,382 putative cDNA sequences as identified from the whole-genome sequence by reciprocal BLAST analysis using a minimum sequence identity threshold of 90% (Kent, 2002). In an alignment of our transcriptome data with these two databases, the 10^−5^ cutoff was used. Matches were found for all but 3450 sequences in the alignment with the published transcriptome ESTs, and 12,766 sequences in the alignment with the whole genome coding sequences and thus, we concluded that our transcriptome data was of high quality and achieved good coverage of the genome. Given the heat inducible characteristic and good genomic coverage of our transcriptome, we opted to use this material as a reference sequence by which to assess the expression profiles of transcripts responding to heat stress. As an additional check, we compared the expression analysis results obtained using the whole-genome sequence of carnation as the reference material with those obtained using our own transcriptome, and found no significant differences (data not shown).

### High temperature affected expression of genes involved in stress responses

Heat can denature proteins, leading to cytotoxicity through the formation of aggregates (Mittler et al., 2011). Protein homeostasis in cells experiencing heat stress may be maintained by the interaction of chaperones with the stress-denatured proteins, in order to prevent their aggregation and malfolding (Qu et al., 2013). It is therefore not surprising that the most strongly upregulated heat induced genes are typically those encoding molecular chaperones such as heat shock proteins (Scarpeci et al., 2008).

Heat shock proteins are a class of functionally related proteins involved in the folding and unfolding of other proteins (Kotak et al., 2007). The expression of this gene group is well documented as rising when cells are exposed to elevated temperatures or other stress types (Agarwal et al., 2003; Kant et al., 2008; Reddy et al., 2011; Chauhan et al., 2012). In this study, numerous heat shock proteins were found to be upregulated after exposure to heat stress, using a threshold expression change of 16-fold (Additional file 2). These responsive genes included six members of the HSP40 family. The HSP40 family plays a role in regulating the ATPase activity of HSP70 by interacting with the HSP70 J domain. Eighteen *HSP70* genes were also found to be upregulated. These genes have been reported to be involved in protein folding and unfolding, thereby providing thermo-tolerance to cells when exposed to heat stress (Hartl, 1996; Sung and Guy, 2003; Su and Li, 2008). Three members of the HSP90 family and one member of the HSP100 family were also upregulated (Additional file 2). In addition, the expression levels of 15 small heat shock proteins were highly induced.

### Heat shock transcription factors may play an important role in response to heat stress

The molecular pathways leading to Hsp expression are not entirely understood, but are reported to involve temperature perception and multiple signal transduction pathways (Xiong and Zhu, 2001; Chinnusamy et al., 2004; Penfield, 2008). These processes lead to the activation of various Hsf proteins that then induce the expression of heat shock genes by binding to the heat shock element (Kotak et al., 2007).

The *LeHsfA1a* gene in tomato appears to have a unique function as the master regulator for acquired thermo-tolerance. There appears to be no substitution of function by any other Hsf, as knock-down of *LeHsfA1a* expression practically eliminates HS-induced synthesis of Hsfs A2 and B1, as well as that of chaperones (Mishra et al., 2002; Baniwal et al., 2004; Von Koskull-Doering et al., 2007). This critical tomato gene is responsible for the initial triggering of the HS response and later on, by interaction with Hsfs A2 and B1 in a functional triad, affects different aspects of the HS response and recovery (Mishra et al., 2002). In *Arabidopsis*, no comparable role as master regulator could be identified for any of the four *AtHsfA1* genes. However, transcriptome analysis of double KO mutants of the four *AtHsfA1* genes has indicated that the encoded Hsfs have specific roles in the HS-induced transcription of various subsets of genes. These induced subsets not only include genes encoding small heat shock proteins (sHsps) Hsp70 and Hsp101, but also genes encoding some Hsfs such as HsfA2, HsfA7a, HsfB1, and HsfB2a, as well as genes encoding HS-induced metabolic enzymes such as inositol-3-phosphate synthase2 (Ips2) and galactinol synthase 1 (GolS1) (Liu et al., 2011). In this study, we also found that the expression levels of carnation *HsfA1, HsfA2*, and *HsfB1* were increased during heat stress.

In *Arabidopsis*, the induction of *HsfA3* expression in response to drought and HS has been shown to be dependent on the DREB2A transcription factor (dehydration-responsive element binding protein 2A) (Schramm et al., 2008). In accord with this, we found in our study that carnation *DREB2A* (*Dc_15272*) showed elevated expression at HS_0.5h and HS_2h, although this had decreased by HS_12h. Correspondingly, *DcHsfA3* (*Dc_9611*) exhibited a higher transcriptional level at HS_2h, which is consistent with the premise that *DcDREB2A* regulates the expression of *DcHsfA3*.

### ROS-scavenging genes' expression changes to maintain cellular homeostasis

Under heat stress, excessive ROS accumulation can lead to the damage of cell membranes, DNA and proteins and hence, the antioxidant enzyme system of plants is enhanced in response to increased ROS levels (Miller et al., 2008; Golldack et al., 2014). In our study, we detected an increase in ROS (Figure 6) and identified several genes encoding antioxidants including, ascorbate peroxidase, AOX, thioredoxin, and glutathione S-transferase which were up-regulated by short-term high temperature stress.

APX is the major H_2_O_2_-reducing peroxidase and is an integral component of the glutathione–ascorbate cycle which has been identified as an important antioxidant mechanism in plants (Ishikawa and Shigeoka, 2008). In *Arabidopsis*, expression of the APX gene family is dependent on heat stress thereby, linking the heat stress response with oxidative stress and stress tolerance (Panchuk et al., 2002; Suzuki et al., 2013). Thioredoxins are proteins that act as antioxidants by facilitating the reduction of other proteins by cysteine thiol–disulfide exchange. They play important roles, not only in redox regulation, but also in the cellular redox signaling network (Arner and Holmgren, 2000). Our study found that two genes encoding thioredoxin were induced by heat stress. This result is in agreement with a previous study in tobacco where, cytosolic APX and thioredoxin peroxidase appeared to be the dominant antioxidants present during heat shock (Rizhsky et al., 2002).

The alternative oxidase (AOX) can oxidize ubiquinol, diverting electrons from the primary cytochrome c pathway in an energetically unproductive process within plant mitochondria to protect against ROS production (Vanlerberghe et al., 2009). In our study of carnation, the expression of mitochondrial AOX was elevated during heat shock, indicating its role in the defense of plant tissues against mitochondrial-generated ROS. In addition, three transcripts encoding glutathione S-transferase (GST) were identified amongst the up-regulated genes. The function of GST has traditionally been considered to be the detoxification of electrophiles by glutathione conjugation (Strange et al., 2001). In this respect, our study agrees with reports of the response of *Saccharina japonica* to high temperature (Liu et al., 2014) and of *Arabidopsis* to heat and drought conditions (Rizhsky et al., 2002) where, GST was also up-regulated by the stress conditions.

### Signal cascades leading to HS induced transcriptional regulation

Previous studies have shown that a large proportion of HS-upregulated transcripts encode heat-induced chaperones which have an important role as part of the HSP-based protection mechanism (Rizhsky et al., 2004; Larkindale and Vierling, 2008; Liu et al., 2008; Qin et al., 2008; Meiri and Breiman, 2009; Perez et al., 2009). Multiple signaling pathways are implicated in the HS response although, all of the putative pathways are thought to activate a similar set of HS-regulated genes in order to enhance thermo-tolerance (Kotak et al., 2007; Saidi et al., 2009; Che et al., 2010; Mittler et al., 2012).

Heat is sensed at the plasma membrane, causing a transient opening of Ca^2+^ channels (Garg et al., 2014) and, thereby, allowing a specific inward flux of extracellular Ca^2+^ ions from the apoplast into the cytoplasm (Saidi et al., 2009). It is postulated that this influx of Ca^2+^ may bind CaM3 (calcium modulated protein) (Liu et al., 2005) thereby activating multiple HS transcriptional regulators such as, HSFs, MBF1c, RBOHS, WRKY, and DREB (Li et al., 2010; Suzuki et al., 2011). In this study, we found that a putative carnation CaM3 gene (*Dc_84559*) showed some degree of up-regulation at each of the three heat treatment time points, although with the highest expression levels detected at HS_0.5h. Similarly, the up-regulation expression profiles of *MBF1c* (*Dc_15682*) and *HSFA2* (*Dc_83420*) transcripts also showed a peak at HS_0.5h with levels decreasing steeply as the time of heat treatment was extended. By contrast, the raised levels of *WRKY* mRNAs (*Dc_42073* and *Dc_68482*) remained fairly stable across the three treatment time points. We suggest that the application of a relatively long period of heat stress before the first sampling time point (i.e., 0.5 h) may account for the lack of evidence in our study of a signal cascade between *CaM3, MBF1c*, and *HSFA2* genes.

In the cytosol and ER, high temperature exposure may trigger the UPR (unfolded protein response) (Mittler et al., 2012). In *Arabidopsis*, two types of the UPR signaling pathway have been reported: one involves two ER membrane-associated transcription factors (bZIP17 and bZIP28), and a second is associated with a dual protein kinase (RNA-splicing factor IRE1) in combination with an ER chaperone (binding immunoglobulin protein, BiP) and its target RNA (bZIP60). In contrast to the ER UPR, the cytosolic UPR of plants appears to be regulated primarily by HSFA2 (Che et al., 2010; Deng et al., 2013; Howell, 2013). In this study, carnation *IRE1* (*Dc_11861*), *bZIP28* (*Dc_55183*), and *BiP* (*Dc_8485* etc.) displayed significantly up-regulated expression levels, while the level of *bZIP17* (*Dc_44881*) mRNA was also slightly increased, suggesting that a cytoprotective mechanism was initiated in carnation leaves subjected to heat stress in order to mitigate the accumulation of unfolded proteins. Expression of carnation *bZIP60* (*Dc_60270*) was not observed in this study, and this remains a subject for future research.

In *Arabidopsis*, the nuclear protein ARP6 regulates a global response to ambient temperature, in part by modulating the nucleosome occupancy of H2A.Z, and a functional ARP6 allele accounted for almost half of the ambient temperature transcript responses (Wigge and Kumar, 2010). In our study, the mRNA level of the deduced carnation *ARP6* gene *(Dc_57637*) was depleted at the three heat treatment time points, so indicating that a histone sensor may also be present within the nucleus of carnation cells and have an active role in the thermo-responsiveness of carnation tissues when subjected to heat stress.

## Materials and methods

### Sample preparation

The aseptically-grown seedlings of *D. caryophyllus* cultivar “Fancy” were employed in all experiments. Following initial culturing for 40 days, the plants were placed in an incubator with 90% relative humidity and an illumination intensity of 120 μmol m^−2^ s^−1^, with various temperature regimes used to provide HS treatments. Total RNA was extracted from leaves using the Trizol reagent (TaKaRa, Inc., Dalian, China) according to the supplier's instructions, treated with RNAase-free DNAase and re-purified with the RNeasy kit (Qiagen, Valencia, CA, USA) according to the manufacturer's protocol. Variously treated plant materials were used as sources of RNA in order to generate the cDNA libraries employed for Illumina HiSeq™ 2000 runs. Thus, for transcriptome sequencing, a pooled RNA sample was created using equal amounts of RNA extracted from leaves of plants subjected to either 42°C HS for 0, 0.5, 1, or 2 h, or 46°C HS for 0.5, 1, or 2 h. For gene expression profiling, leaves were harvested at time points 0, 0.5, 2, and 12 h after the start of the 42°C heat treatment. For real-time quantitative PCR analysis, leaves were harvested at time points 0, 0.5, 1, 2, 4, 8, and 12 h after the start of the 42°C heat treatment. With the exception of samples for transcriptome sequencing, all analyses were conducted using the results from three independently replicated experiments. All harvested samples were immediately frozen in liquid nitrogen and stored at −80°C until required for further processing.

### cDNA library preparation and sequencing

Poly(A) mRNA from the total RNA was purified using oligo(dT) beads. Following purification, mRNA was fragmented into small pieces using divalent cations. The first cDNA strand was synthesized using random hexamer primers for reverse transcription with cleaved mRNA fragments serving as templates. The second strand cDNA was synthesized using DNA polymerase I (Qiagen), incubated with RNase H (Invitrogen), dNTPs and buffer. Following end repair, a single “A” was added to the blunt end, and then adaptors were added to the purified double stranded cDNA fragments. The products (150–300 bp) are purified and enriched with PCR (15 cycles) to create the final cDNA library. The PCR amplification products for transcriptome sequencing were sequenced with Illumina HiSeq™ 2000 sequencing platform, using paired-end reads with a length of 2 × 100 nucleotides, and this was carried out at Beijing BerryGenomics Co. Ltd. (Beijing, China). The PCR amplification products for gene expression profiling were sequenced with Illumina HiSeq™ 2000 at Genergy BioTechnology Co. Ltd. (Shanghai, China).

### Sequence assembly and annotation

Following sequencing, primer and adaptor sequences were removed from the database so retaining only high quality sequences of the original nucleotide fragments. A RNA-Seq quality control assessment was carried out based on evaluations of transcriptome mapping and gene-body coverage of the original data. The resulting reads were subsequently analyzed according to the RNA-Seq project analysis module (Haas and Zody, 2010). Reads were assembled using Trinity (Grabherr et al., 2011). Reads were assembled by overlapping the fragments from the original data, and the longest assembled reads were defined as contigs. Multiple contigs were assembled into scaffolds. Those sequences which could be identified as genes encoding proteins were defined as unigenes. The predicted amino acid sequences encoded by such unigenes were aligned with sequences in NCBI Nr database, the Swiss-Prot Protein database, gene ontology (GO) analysis and the KEGG pathway database, using BLASTx searches with an *E*-value threshold of 1 × 10^−5^. Protein function information was predicted from the annotations corresponding to the most similar proteins found in the databases. GO enrichment analysis (on the basis of biological process, cellular component or molecular function) was conducted using hypergeometric distribution algorithms and the Fisher exact test. GO terms were assigned to each unique gene based on the GO annotation of the corresponding homologs in the database (Ye et al., 2006). Using the Kyoto Encyclopedia of Genes and Genomes (KEGG) pathway database (a record of networks of molecular interactions in cells) (Kanehisa and Goto, 2000), we mapped the identified carnation sequences to specific biochemical pathways.

### Gene expression profiling

The measured transcript levels were statistically analyzed to test the significance of the differentially expressed genes between samples. Gene expression levels were calculated according to the metric FPKM (Fragments Per Kilobase of transcript per Million fragments mapped) (Mortazavi et al., 2008) and analysis was performed using Cufflinks software (Trapnell et al., 2012). To test whether the gene expression levels responded significantly to heat shock, we compared the log ratio of HS and control samples. Differentially expressed genes (DEGs) were identified according to statistically significant differences with the threshold of *P* < 0.05 (Audic and Claverie, 1997). All such DEGs were further annotated by GO and KEGG pathway enrichment analyses.

### Real-time quantitative PCR analysis

Total RNA was extracted using EASYspin rapid plant RNA extraction kit (Aidlab Biotechnologies CO. Ltd. RN09). The integrity and quality of RNA was checked by agarose gel electrophoresis and spectrophotometry. A 1 μg sample of total RNA was used to produce 20 μl cDNA for real-time quantitative PCR, for which we used the PrimeScript® RT reagent Kit with gDNA Eraser (Perfect Real Time), (TaKaRa, Code: DRR047A, Japan).

To validate our expression profile data, we selected 10 genes showing significant expression changes according to this analysis and performed real-time quantitative PCR. Gene specific oligonucleotide primers (Additional file 4) for real-time quantitative PCR were designed using Primer Premier 5. Primers were designed for amplicons, covering approximately 150 bp over a non-conservative region of each gene, and these were tested to ensure that no primer-dimer products resulted in single discrete band amplification. A carnation *GAPDH* (*Glyceraldehyde-3-phosphate dehydrogenase*) gene was used as the control transcript in each reaction.

We used the standard curve method to validate the expression profile data. Standard curve samples were prepared as follows: a mixture comprising equal quantities of cDNA taken from all samples of reverse transcription reactions formed the first point of the standard curve, and this mixture was serially diluted 10-, 100-, 1000-, and 10,000-fold to generate the seconds, third, fourth and fifth points, respectively. Polymerase chain reactions were performed in 96-well plates in the ABI Prism 7500 Fast Real-Time PCR System (Applied Biosystems, USA) using SYBR® *Premix Ex Taq*™ II (Tli RNaseH Plus) (Takara, Japan). Real-time quantitative PCR products were amplified using 10 μl SYBR® *Premix Ex Taq* II (Tli RNaseH Plus) (2×), 0.8 μl PCR forward and reverse primer (10 μM), 0.4 μl ROX Reference Dye II (50×), 2 μl cDNA (diluted 10-fold) template from the RT reaction, and 6 μl sterile distilled water to give a final volume of 20 μl. PCR amplification conditions were as follows: 95°C for 30 s followed by 40 cycles of 95°C for 3 s, 60°C for 30 s.

To evaluate the overall specificity of the PCR reaction, a negative control lacking cDNA template was run alongside each analysis. For each gene, three biological replicates and three technical replicates were conducted for each time point. The quantification of gene expression was calculated using the *Relative Expression Software Tool-Multiple Condition Solver REST-MCS © - version 2* software, and data are shown as mean values ± SD (standard deviation).

### Detection and quantification of ROS

ROS accumulation in carnation leaves under heat shock was detected according to Fukao et al. (2011). Plants grown under normal (20°C) temperature conditions were used as control material. Samples for ROS detection were collected at 0, 0.5, 2, and 12 h following the start of a 42°C heat treatment. To visualize superoxide accumulation, the leaves from heat-treated or untreated (control) carnation plants were excised and immediately placed into a 0.5 mg/mL NBT solution in 10 mM potassium phosphate buffer, pH 7.6, and incubated at 25°C for 3 h in darkness. For hydrogen peroxide detection, excised aerial vegetative samples were treated with 1 mg/mL DAB in 50 mM Tris acetate buffer, pH 5.0, and incubated at 25°C for 24 h in darkness. Following staining, the uppermost leaf of each plant was boiled in 95% (v/v) ethanol for 20 min to remove chlorophyll, and rehydrated by incubating in 40% (v/v) glycerol for 16 h at 25°C. Each experiment was repeated using at least seven different plants, and representative images are presented.

### Conflict of interest statement

The authors declare that the research was conducted in the absence of any commercial or financial relationships that could be construed as a potential conflict of interest.
