# Supplementary material for: Identification of heat-responsive genes in carnation (Dianthus caryophyllus L.) by RNA-seq
Source: Front Plant Sci. 2015 Jul 14;6:519. doi: 10.3389/fpls.2015.00519 (PMC4500917; doi:10.3389/fpls.2015.00519)
Supplement: Supplementary file 10 [file Presentation1.PPT]

## Slide 1
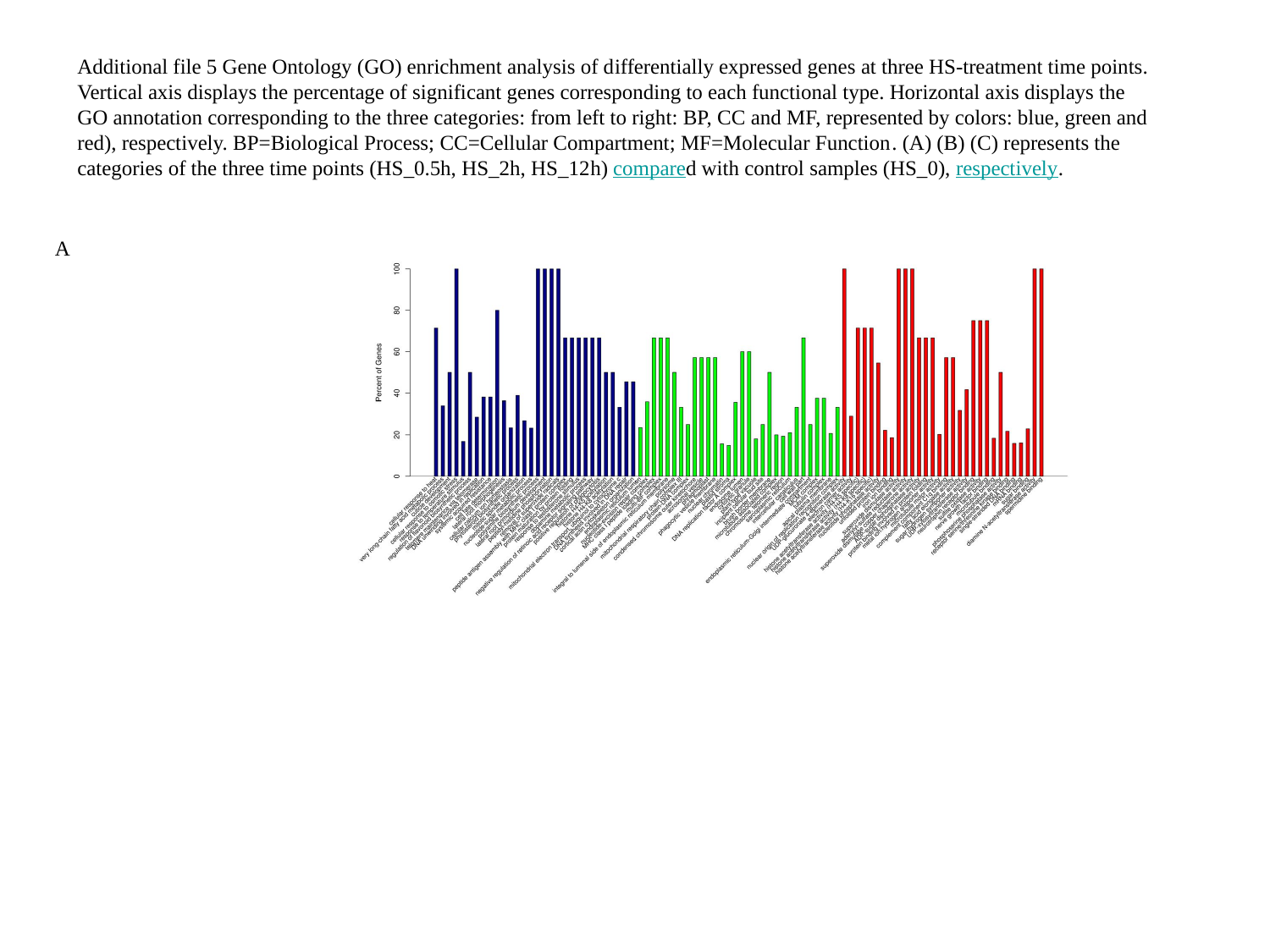

Additional file 5 Gene Ontology (GO) enrichment analysis of differentially expressed genes at three HS-treatment time points. Vertical axis displays the percentage of significant genes corresponding to each functional type. Horizontal axis displays the GO annotation corresponding to the three categories: from left to right: BP, CC and MF, represented by colors: blue, green and red), respectively. BP=Biological Process; CC=Cellular Compartment; MF=Molecular Function. (A) (B) (C) represents the categories of the three time points (HS_0.5h, HS_2h, HS_12h) compared with control samples (HS_0), respectively.
A

## Slide 2
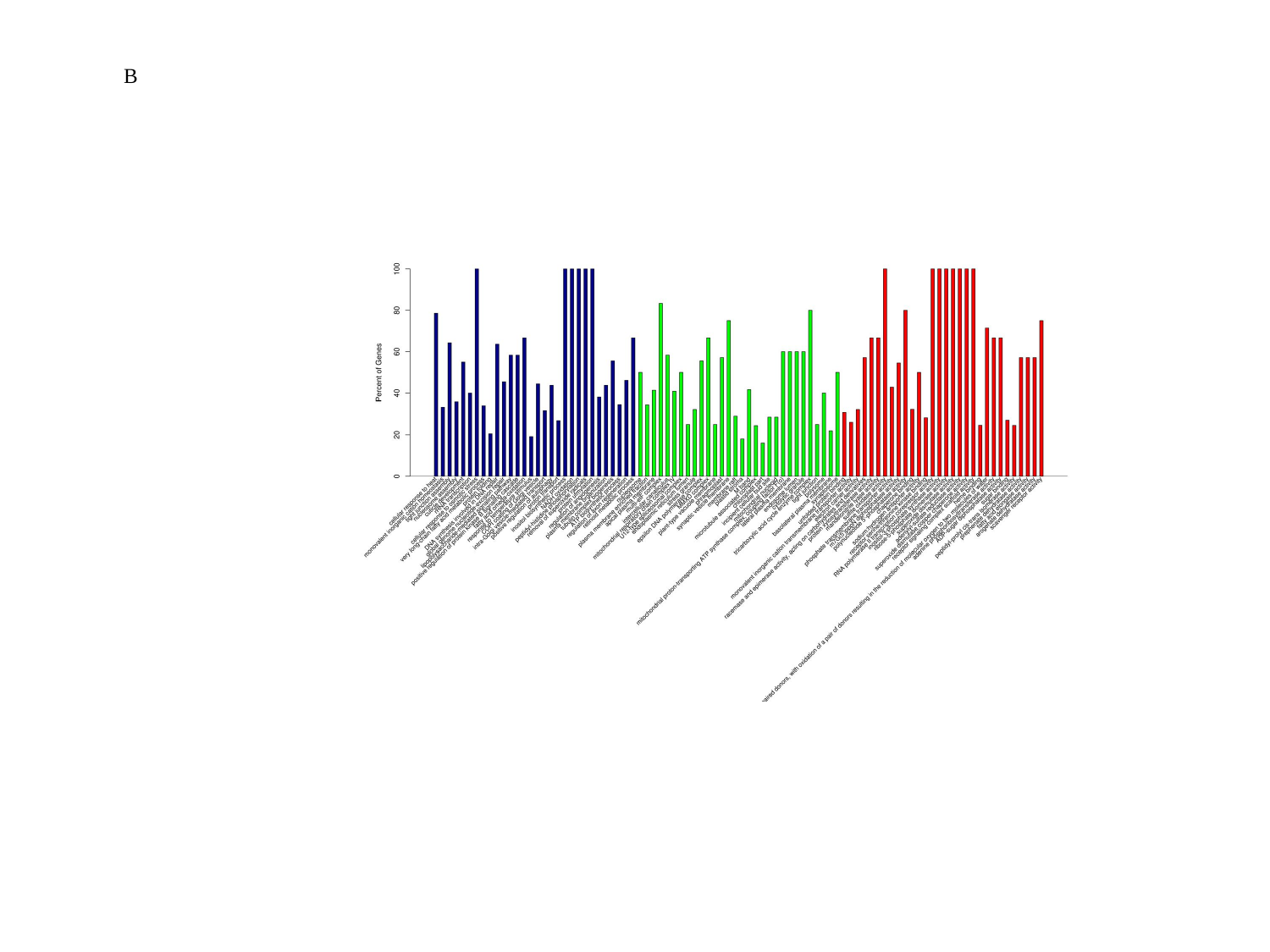

# B

## Slide 3
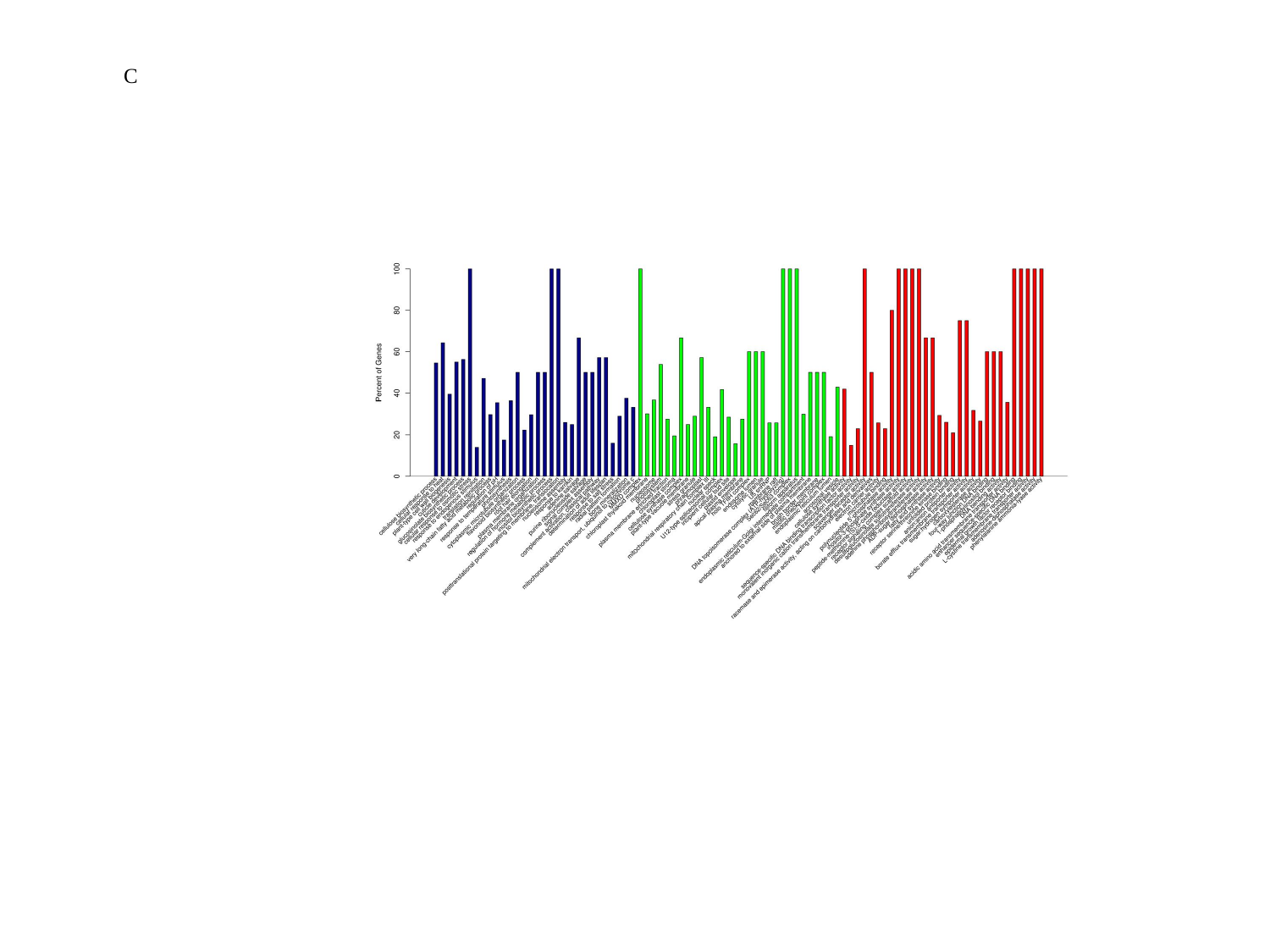

# C
